# Integrating Infant Safe Sleep and Breastfeeding Education Into an App in a Novel Approach to Reaching High-Risk Populations: Prospective Observational Study

**DOI:** 10.2196/65247

**Published:** 2025-01-14

**Authors:** Tamar Krishnamurti, Rachel Moon, Rudolph Richichi, Rachel Berger

**Affiliations:** 1 School of Medicine, University of Pittsburgh, Pittsburgh, PA, United States; 2 School of Medicine, University of Virginia, Charlottesville, VA, United States; 3 Statistical Analysis Management Consultants, Alexandria, VA, United States; 4 Child Advocacy Center, Department of Pediatrics, UPMC Children's Hospital of Pittsburgh, Pittsburgh, PA, United States; 5 Pennsylvania Office of Children, Youth and Families, Harrisburg, PA, United States

**Keywords:** SIDS, infant death, sleep, sudden infant death, US, United States, infant, infancy, baby, prenatal, safe sleep, breastfeeding, infant care, pregnancy, app, randomized controlled study, TodaysBaby, mobile health, mHealth, smartphone

## Abstract

**Background:**

Sudden unexpected infant death (SUID) is a leading cause of death for US infants, and nonrecommended sleep practices are reported in most of these deaths. SUID rates have not declined over the past 20 years despite significant educational efforts. Integration of prenatal safe sleep and breastfeeding education into a pregnancy app may be one approach to engaging pregnant individuals in education about infant care practices prior to childbirth.

**Objective:**

This study aims to assess whether pregnant individuals would engage with prenatal safe sleep and breastfeeding education provided within a pre-existing pregnancy app. Secondary objectives were to compare engagement among those at high and low risk of losing an infant to SUID and to assess the importance of end user push notifications for engagement.

**Methods:**

This prospective observational study was conducted from September 23, 2019 to March, 22 2022; push notifications were removed on October 26, 2021. TodaysBaby (University of Virginia, Boston University, and Washington University), a mobile health program in which safe sleep and breastfeeding video education was originally provided via texts, was embedded into the MyHealthyPregnancy app (Naima Health LLC). Pregnant mothers who received prenatal care within the University of Pittsburgh Medical Center hospital system were randomized to receive either safe sleep or breastfeeding education beginning at the start of the third trimester of pregnancy and ending 6 weeks post partum. Pregnant persons were designated as high risk if they lived in the 5% of zip codes in Allegheny County, Pennsylvania with the highest rates of SUID in the county. The primary outcome was engagement, defined as watching at least 1 video either in response to a push notification or directly from the app’s learning center.

**Results:**

A total of 7572 pregnant persons were enrolled in the TodaysBaby Program—3308 with push notifications and 4264 without. The TodaysBaby engagement rate was 18.8% with push notifications and 3.0% without. Engagement was highest in the initial weeks after enrollment, with a steady decline through pregnancy and very little postpartum engagement. There was no difference in engagement between pregnant persons who were low and high risk. The most viewed videos were ones addressing the use of pacifiers, concerns about infant choking, and the response of the body to the start of breastfeeding.

**Conclusions:**

Integrating safe sleep and breastfeeding education within a pregnancy app may allow for rapid dissemination of infant care information to pregnant individuals. Birthing parents at high risk of losing an infant to SUID—a leading cause of infant death after 1 month of age—appear to engage with the app at the same rates as birth parents who are at low risk. Our data demonstrate that push notifications increase engagement, overall and for those in high-risk zip codes where the SUID education is likely to have the most impact.

## Introduction

Sudden unexpected infant death (SUID) is a leading cause of death among US infants, with nonrecommended sleep practices reported in most of these deaths [1]. After a major decrease in the SUID rate immediately after the American Academy of Pediatrics released its 1992 recommendation that infants be placed on their backs to sleep, the rate has plateaued despite extensive educational efforts [2,3]. There continue to be racial and socioeconomic disparities in SUID, with rates higher among Black infants and infants who live in poverty [4-6]. The disparity can be marked, for instance, in Allegheny County, Pennsylvania in 2017‐2022; 35% of all SUID occurred in 5.6% (7/124) of zip codes (Berger R, MPH, MD, unpublished data, personal communication, May 2024).

Parental education in the newborn nursery has been an important approach to reducing the risk of SUID [7-9]. Information about modifiable risk factors for SUID, including prone or side sleep position, not breastfeeding, sharing a sleep surface, use of soft bedding, and exposure to nicotine/smoking, alcohol, drugs, and illicit substances, when routinely incorporated into newborn education, can change parental practice [4,10]. In one 4-armed randomized controlled trial of hospital quality improvement (QI) and mobile health (mHealth) in 16 US hospitals, new parents were enrolled in an mHealth program (TodaysBaby; University of Virginia, Boston University, and Washington University). The videos in TodaysBaby were designed with parental input from mothers of young infants (our target population); mothers were involved design of the videos and provided suggestions before and during video production and provided feedback after video production [9]. Participants in the randomized controlled trial were randomized to receive emails or texts with links to short (<2 min) videos about either breastfeeding or safe sleep for 60 days. The videos were aimed at changing attitudes and dispelling misconceptions about safe sleep and breastfeeding. In this study, parents who received only safe sleep messaging were 10 percentage points more likely to use safe sleep practices than parents who received no safe sleep messaging. Additionally, racial and ethnic differences in reported safe sleep practices were eliminated in those who received safe sleep messaging. To our knowledge, this is the only randomized controlled trial evaluating video education as an approach to improving safe sleep practices.

The MyHealthyPregnancy (MHP; Naima Health LLC) app is a commercially available evidence-based patient-facing smartphone app and provider-facing portal developed to monitor and model individual risk during pregnancy and provide easy, actionable feedback to patients. The app also offers connection to relevant resources both within and outside the health care system and notifies the individual’s care team if a critical risk (eg, preterm labor and suicidal ideation) is reported through the app. The primary goal of the app is to reduce unwanted outcomes both during pregnancy and in the early period after delivery through more timely risk detection and intervention [11-14]. End users were involved in the design, development, and deployment of the technology and the design approach was grounded in person-centered frameworks to advance reproductive health equity [11,15].

The primary objectives of this study were to assess whether the TodaysBaby educational program could be integrated into the MHP app, whether users of the app would engage with the TodaysBaby content, and whether there were differences in knowledge and attitudes related to safe sleep among pregnant persons who were assigned to the safe sleep versus breastfeeding education. A secondary objective was to assess whether parents of infants at the highest risk of SUID—as defined by zip code—were similar in their rates of engagement, knowledge, and attitude compared with those living in low-risk zip codes. TodaysBaby was the first infant-specific education to be integrated into the MHP app, which is otherwise entirely focused on pregnant persons’ health and pregnancy.

## Methods

### Overview

Providers at prenatal clinics in the University of Pittsburgh Medical Center (UPMC) health system could prescribe the MHP app (iOS version 1.4.7, Android version 1.8) to pregnant patients at their first prenatal appointment as part of a prenatal care QI initiative sponsored by UPMC and at no cost to the patient. The internal protocol for prescribing MHP was to send a text-based invitation with a unique web link to the patient’s phone, which allowed app users to download the app from the Android or Apple app store. App users electronically consented to share identifiable data with their health care provider and anonymized aggregate data for research. An additional specific consent was provided for enrolling in TodaysBaby. Participants did not receive financial compensation for app use or TodaysBaby participation. At the time of consent, patients were randomly assigned to the safe sleep or breastfeeding group in a 2:1 ratio. High-risk patients—defined by zip code as described below—were enrolled in a 3:1 ratio to the safe sleep group.

All participants were recruited during pregnancy at prenatal clinics. While we recognize that pregnant individuals and parents may be of any gender and that transgender men and gender-nonbinary people may also give birth, we did not collect additional information about biological sex or gender identity from participants. We refer to participants as pregnant persons throughout the manuscript.

MHP users completed an initial onboarding process in which they completed questions about demographics, medical history, and baseline risk factors. Throughout the course of their pregnancy, app users were offered the opportunity to answer questions about their experiences and symptoms through app-embedded screeners, questionnaires, and open-ended text entries. Starting at 32 weeks of pregnancy (based on the due date entered by the user), participants started receiving texts with links to videos (“push notifications” or “pushes”) at predefined intervals. The decision to start TodaysBaby at 32 weeks was a change from the original TodaysBaby study, in which the education started at the birth of the infant [9]. We chose not to start earlier than 32 weeks to minimize the likelihood of pregnancy loss among study participants.

There were 21 pushes with 18 videos for each group (safe sleep and breastfeeding): 14 pushes in the prenatal period and 7 in the postnatal period. A total of 12 short quizzes about intentions related to sleep and breastfeeding were pushed at similar intervals. All videos were also available to all users in both groups at any time in the app’s learning center (LC), which was a section of the app providing educational articles and video links on a variety of pregnancy-related topics.

The video topics for both the safe sleep and breastfeeding groups are summarized in Textbox 1.

Textbox 1. **Video topics for the safe sleep and breastfeeding groups.**

**Safe sleep**

- Importance of sleep position
- Choking and sleep position
- Importance of sleep space
- Bed-sharing
- Handling advice from others
- Mattress safety
- Soft bedding
- Feeding baby in bed
- Pacifiers
- Dangers of smoking
- Infant sleep patterns

**Breastfeeding**

- Importance of breastfeeding
- How to start breastfeeding
- How often to breastfeed
- Hunger cues
- What to do if baby is always hungry
- Benefits of breast milk
- How long it takes for milk to come in
- Latching on
- Avoiding breast discomfort/pain
- Dealing with fussy baby
- Getting support from others
- Economics of breastfeeding
- Breastfeeding when returning to work

On October 26, 2021, the push notifications and videos were removed; the videos remained in the LC for pregnant persons who downloaded the MHP app.

### Ethical Considerations

The project was approved by the UPMC QI Committee (reference number 1613). App users consented the publication of anonymized aggregate data during app onboarding. An additional consent was provided during app onboarding to a subset of MHP users to opt in to participation in the TodaysBaby program.

### Statistical Analysis

The number of videos watched via push notification or from the LC was aggregated. Data from the push notifications indicated that some videos were possibly clicked, so the aggregation was done once for definitely clicked videos and separately to include both and possibly clicked videos. Partial viewings of a video were counted as a viewing of that video. Engagement was defined as watching at least 1 video either in the LC or in response to a push notification. A binary indicator of high- versus low-risk zip codes was created.

Descriptive statistics such as frequency and percentages were used to examine demographic factors for the entire sample as well as the high- and low-risk groups. Individual chi-square tests were used to examine 2 × 2 combinations by group (safe sleep vs breastfeeding), engagement, high-risk zip codes, and frequency of video viewership. A standard of 5% probability of type 1 error was used.

## Results

### Overview

Between September 23, 2019, and October 26, 2021, 43% (3635/8453) of pregnant persons who were prescribed the MHP app enrolled. Of these, 91% (n=3308) consented to receive TodaysBaby content—2407 in the sleep group and 901 in the breastfeeding group. There were no demographic differences between those who did or did not enroll in the MHP app [13]. The demographics of participants who enrolled in TodaysBaby are summarized in Table 1.

**Table 1.** Demographics of MyHealthyPregnancy app users who consented to TodaysBaby.

		Overall users (n=3308)	Pregnant person living in a higher SUID^a^ rate area in Allegheny County (n=297)	Pregnant person living in a lower SUID rate area in Allegheny County (n=1562)
Maternal age (years), mean (SD)		29.8 (5.4)	30.3 (5.8)	30.7 (5.1)
Income (US $), n (%)				
	Under 10,000	282 (8.9)	43 (15.4)	105 (7.0)
	10,000-14,999	147 (4.6)	18 (6.4)	56 (3.7)
	15,000-19,999	107 (3.4)	11 (3.9)	39 (13.3)
	20,000-24,999	137 (4.3)	18 (6.4)	48 (3.2)
	25,000-34,999	233 (7.3)	21 (7.5)	86 (5.7)
	35,000-49,999	274 (8.6)	21 (7.5)	103 (6.9)
	50,000-69,999	351 (11.0)	28 (9.4)	140 (9.3)
	70,000-100,000	640 (20.1)	43 (15.4)	306 (20.4)
	>100,000	1011 (31.8)	77 (27.5)	618 (41.2)
Parity–Nulliparous, n (%)		1793 (54)	166 (56)	885 (57)
Maternal education, n (%)				
	Bachelor degree or higher	1881 (57)	158 (53)	1043 (67)
	Associate degree	356 (11)	29 (10)	137 (9)
	Grade school, some high school, high school, or GED^b^	1025 (31)	101 (34)	362 (23)
	Missing	46 (1)	9 (3)	20 (1)
Smoked tobacco, n (%)		219 (6.6)	20 (6.7)	70 (4.5)
Vaped, n (%)		64 (1.9)	3 (1.0)	24 (1.5)
Used marijuana, n (%)		104 (3)	18 (6.1)	39 (2.5)
Maternal race, n (%)				
	White	2608 (79)	164 (55)	1147 (73)
	Black	351 (11)	91 (31)	210 (13)
	East or South Asian	149 (5)	12 (4)	103 (7)
	Other	176 (5)	25 (8)	90 (6)
	No response	24 (0)	5 (2)	12 (1)

a SUID: sudden unexpected infant death.

b GED: General Educational Development.

### Engagement

#### Video Watching in Response to a Push Notification

Overall, 11% (368/3308) of all participants clicked on at least one of the push notifications they received. Of those who clicked, 53% (194/368) clicked only 1, 26% (94/368) clicked on 2 unique notifications, and the remaining 21% (80/368) clicked on 3 or more unique push notifications (maximum 20 push notifications). An additional 36 participants clicked on at least 1 push notification but because they closed the app immediately after clicking on the notification, it is not possible to be sure whether they read the information provided. If the possible clicks are included, then 12.2% (404/3308) of all participants clicked on a push notification. By comparison, 9.0% (300/3308) of users clicked on at least 1 monthly mental health push notification which was part of the MHP app but not part of TodaysBaby.

#### Video Watching From the LC

Overall, 10.4% (347/3308) watched at least 1 video directly from the LC and not in response to a push notification; 6.2% (206/3308) watched at least 1 breastfeeding video from the LC, and 7.4% (345/3308) watched at least 1 safe sleep video from the LC.

#### Overall Engagement Rate

Overall engagement, defined as watching at least 1 video either in response to a push notification or from the LC, was 18.8% (623/3308).

#### Video Watching

The frequency with which videos were watched ranged from 1% (35/3308) (“why you should not smoke around your baby”) to 4.7% (154/3308) (“a reminder about pacifiers”). Over 9% (300/3308) watched at least 1 of 3 pacifier-related videos. Over 98% (613/623) of the watched videos were watched only once (Table 2).

**Table 2.** Frequency with which the 10 most popular safe sleep and breastfeeding videos were watched.

Video name	Number of views
A reminder about pacifiers	154
Should I give my baby a pacifier?	106
Will my baby choke on the stomach?	105
Will my body know what to do when I start breastfeeding?	98
Why sleep position matters	97
How often should I feed my baby?	90
What is the safest mattress?	89
How do I know when my baby is hungry	86
What about bedding and bumpers?	84
What makes a baby a good sleeper	70

#### Timing of Video Watching

Timing of video watching during pregnancy could only be assessed for app users for whom the TodaysBaby integration took place during their first pregnancy with the app. The “weeks gestation” field was not consistently accurate for users who had used the app with previous pregnancies because some had not logged the date when the first pregnancy ended.

Of the 3308, 43% (n=1431) users were using the app for the first time. The mean time during gestation, when videos were watched, ranged from 19 weeks of gestation (“How long should I breastfeed?” and “More about pacifiers”) to 32‐33 weeks (“What can I do when my baby is fussy” and “Breastfeeding can save you time and money”). Many of these videos were watched through the LC before the TodaysBaby curriculum was available (at 32 wk of gestation) on the MHP app.

#### Engagement Rate After Removal of Push Notifications

From October 27, 2021 to March 22, 2022, after the push notifications were stopped, of the 4264 participants who downloaded the MHP app, 3.0% (n=127) watched any TodaysBaby videos. Overall, 2.0% (n=85) watched at least 1 breastfeeding video, and 2.0% (n=85) watched at least 1 safe sleep video. The use of the LC was also significantly lower among pregnant persons who did not receive pushes versus those who did (127/4264, 3% vs 347/3308, 10.4%; *P*<.001).

#### Engagement of Participants in High-Risk Zip Codes in Allegheny County

Of the 3308 users, 58% (n=1859) were from Allegheny County; of these, 16% (297/1859) lived in a high-risk zip code and 84% (1562/1859) lived in a low-risk zip code. Demographic characteristics of the TodaysBaby participants from high-risk and low-risk zip codes are found in Textbox 1.

Among those with high-risk zip codes, 219 were randomized to safe sleep and 78 to the breastfeeding group. Overall engagement in this group was 21.5% (62/297), 10.8% (32/297) clicked on at least 1 TodaysBaby push notification, and 12.5% (37/297) watched at least 1 video from the LC. View rates of safe sleep and breastfeeding videos were similar for both high-risk and low-risk groups.

There was no difference in the engagement of users from high-risk and low-risk zip codes, with the push notifications (32/297, 10.8% vs 185/1562, 11.8%; *P*=.60) or the LC videos (182/1562, 11.6% vs 37/297, 12.5%; *P*=.70).

#### Quizzes

Overall, 12.0% (409/3308) of all users took at least 1 of the 12 quizzes. Of those who took at least 1 quiz, 54% (222/409) took only a single quiz. Except for quizzes 1 and 6, the completion rate was very low (Table 3). Quizzes 1 and 6 coincided with the user getting a push notification on the same day related to a change in the stage of fetal development. The push notification on the day of quiz 1 (32 weeks gestation) was “Pregnancy is full of many joys and challenges! Reflect on yours now with your mental health check-in” and the one on the day of quiz 6 (35 weeks gestation) was “Welcome to week 35! Time to put that hospital bag next to your door! Visit MHP for a list of recommended items to pack.” There was no difference in response rate between the safe sleep and breastfeeding groups or between high- and low-risk users.

**Table 3.** Number of responses to each of the 12 quizzes.

Quiz number	Number of responses
1	279
2	32
3	32
4	44
5	48
6	211
7	62
8	34
9	32
10	25
11	1
12	2

The responses to questions in quizzes 1 and 6 demonstrate that app users plan to breastfeed (83.2%), plan for their baby to sleep in a safe location (97.9%), and plan for their baby to sleep on his or her back (94.6%) (Table 4). There was no difference in the quiz responses between groups (safe sleep versus breastfeeding or high versus low risk).

**Table 4.** Responses to quizzes 1 and 6.

Quiz number and quiz question	Response
Q^a^1Ques^b^1: When I first bring my baby home, I plan to breastfeed	83.2% agree or strongly agree
Q1Ques2: When I first bring my baby home, I plan to have him/her sleep in an adult bed	2.1% agree or strongly agree
Q1Ques3: When I first bring my baby home, I plan to place him/her on the side to sleep	5.7% agree or strongly agree
Q1Ques4: When I first bring my baby home, I plan to place him/her on the stomach to sleep	3.9% agree or strongly agree
Q1Ques5: When I first bring my baby home, I plan to place him/her on the back to sleep	94.6% agree or strongly agree
Q1Ques6: When I first bring my baby home, I plan to give him/her a pacifier	34% agree or strongly agree
Q6Ques1: I will be taking a medication or substance that may make me sleep more deeply	10.9% yes or unsure
Q6Ques2: I am currently on prescribed opioids/pain medication/methadone or Subutex/suboxone	1.9% yes
Q6Ques3: I currently use tobacco products	7.6% yes

a Q: quiz.

b Ques: question.

## Discussion

### Principal Findings

This is the first study, to our knowledge, to evaluate the use of a mobile app as a means of delivering safe sleep education to pregnant persons with the goal of addressing the risk of SUID. While mHealth tools have been used to explicitly encourage breastfeeding and other care behaviors, the use of an mHealth tool to address safe sleep is novel [16,17]. Over the past 20 years, the way in which young adults receive information has shifted. Smartphone ownership is ubiquitous among reproductive-aged individuals in the United States, with similar distribution by race and ethnicity [18]. A vast majority of smartphone owners use their phones to access health information, with pregnancy being one of the most popular health app domains [19-21].

In addition to their popularity as an information source, there are several clear advantages to the dissemination of information through a mobile app. An app allows for the dissemination of information much more broadly and quickly than face-to-face education or information via educational pamphlets. It also provides the opportunity for users to obtain the information at a time and place which is convenient for them and it allows for the education to be provided as many times as desired and for it to be provided in the same way each time. Receiving inconsistent information even within a single setting such as a primary care physician’s office or newborn nursery can contribute to misinformation.

Timing of education is also critical. While previous safe sleep and breastfeeding education programs have focused on parents after childbirth, parents make many decisions about sleep and feeding practices during pregnancy. In a study by our group, we found that pregnant mothers often purchased cribs and made decisions about sleep practices in their third trimester (Berger R, MPH, MD, unpublished data, 2021). Much of the safe sleep and breastfeeding education in the health care system takes place in a physician’s office or newborn nursery after birth, which may not coincide with when parents or other caretakers want or need to hear this information. For this reason, we included TodaysBaby within the MHP app starting at 32 weeks, which was a change from the original TodaysBaby study, in which the education started at the birth of the infant [9]. Our data demonstrated that many of the videos in the LC center were watched before any of the app users began receiving the TodaysBaby curriculum. The LC provides the opportunity for pregnant persons to find the information they are most eager and willing to learn and may be most amenable to incorporating new information. As a result of this finding, integration of the TodaysBaby curriculum earlier in the pregnancy is a possible future change.

One of the challenges of safe sleep education provided in a physician’s office or birthing hospital is that there is a set curriculum; it would be very difficult to tailor education to each parent. In a recent study by our group, parents described interactions with their pediatricians related to safe sleep. Most parents reported that their pediatricians asked if they were practicing safe sleep and if they responded they were, there was no further conversation (Sahud H, unpublished data, personal communication, 2024). Here, an additional advantage of app-based content delivery is that the education can be tailored. Our study showed distinct differences in popularity (as determined by number of views) depending on the topic of the videos. This suggests that parents know the specific topics they are interested in or need additional information about (eg, pacifier use) and may not want to spend time learning about other (potentially already-familiar) infant-related topics even though each of the videos was <2 minutes in length.

This study also allowed us to see the natural engagement rate with the TodaysBaby content when deployed outside the context of a controlled study environment in a nonincentivized manner. Our approach allowed for dissemination to a large number of users which would not be possible with an in-person dissemination strategy. Since the field of digital health does not yet have a consensus definition of how to measure and report engagement [22], and very few published studies share engagement rates for specific embedded features and content within a larger intervention (eg, embedding TodaysBaby within the MHP app), it is difficult to interpret our engagement rate of 18.8%. Two studies evaluating specific intervention-embedded content in nonincentivized tool use demonstrated much lower engagement rates (eg, <1% of users completed all modules in 1 mHealth tool; approximately 3.5% completed a single assessment in another) than ours [23,24]. There may be an opportunity to increase engagement by focusing on high-interest video topics and beginning the TodaysBaby curriculum earlier in pregnancy when the overall engagement with the app is higher. Although fewer than half of the videos were watched in response to pushes, when the pushes were removed, the overall engagement dropped from 18% to 3%. Moreover, engagement with quizzes was highest when those quizzes coincided with push notifications containing fetal development information. This suggests that push notifications are critically important to engagement, and push notifications with time-sensitive or high-interest content may be leveraged to engage individuals with aspects of the tool that they would otherwise not seek out. The similar engagement rates between users living in communities with high and low rates of SUID are consistent with other data from the MHP app which has demonstrated that pregnant persons at the highest risk for pregnancy-related complications had similar or higher levels of engagement (Naima Health, unpublished data). This finding has important implications for SUID prevention and potentially for the identification of populations most likely to benefit from access to the MHP app. It is also encouraging that the engagement with videos about maternal health within the MHP app was watched as frequently as videos within the TodaysBaby curriculum since it suggests that mothers are equally willing to watch videos about their baby’s health and their own health.

### Limitations

There are limitations to this study. Those individuals who downloaded the app may not be representative of the larger pregnant population. For example, these individuals may be more comfortable with seeking out information through technology and may not reflect the population in greatest need of the TodaysBaby information. Because of the low response rate for the quizzes, it was not possible to assess whether engagement with the app correlated with changes in safe sleep or breastfeeding intentions. All participants were English-speaking; there is current work on an app that will be in both Spanish and English (Krishnamurti T, unpublished data, personal communication, June 2024).

### Conclusions

Our data demonstrate that it is possible to embed safe sleep and breastfeeding education into an app designed to improve outcomes during pregnancy; that the engagement level is encouraging, especially when compared with other published studies of intervention-embedded education within a health-related app; and that this engagement was equally as high among pregnant persons at the highest risk of an SUID. Using an app to disseminate information allows for widespread and rapid dissemination and provides users with the opportunity to choose which education they are interested in and when they are interested in receiving it. Simply having access to this education—as was available in the app LC—is not sufficient for engagement, and push notifications are critical for engagement. Future research should focus on assessing whether engagement with the app and TodaysBaby curriculum correlates with changes in safe sleep practices.

## Abbreviations

LC: learning center
mHealth: mobile health
MHP: MyHealthyPregnancy
QI: quality improvement
SUID: sudden unexpected infant death
UPMC: University of Pittsburgh Medical Center
